# The Preparation and Identification of a Monoclonal Antibody against Domoic Acid and Establishment of Detection by Indirect Competitive ELISA

**DOI:** 10.3390/toxins9080250

**Published:** 2017-08-17

**Authors:** Abdullah F. U. H. Saeed, Sumei Ling, Jun Yuan, Shihua Wang

**Affiliations:** Fujian Key Laboratory of Pathogenic Fungi and Mycotoxins, Key Laboratory of Biopesticide and Chemical Biology of the Education Ministry, School of Life Science, Fujian Agriculture and Forestry University, Fuzhou 350002, China; abdullahfarhan@hotmail.com (A.F.U.H.S.); Lsmpu2008@163.com (S.L.); yjmail2008@126.com (J.Y.)

**Keywords:** domoic acid, monoclonal antibody, cell fusion, hybridoma technology, ic-ELISA

## Abstract

Domoic acid (DA) is a potent toxin, marine biotoxin, and primarily produced by *Pseudo-nitzschia*. The DA hapten was coupled with bovine serum albumin (BSA), and ovalbumin (OVA) as carrier proteins. DA-BSA conjugate was used as immunogen and DA-OVA as coating antigen. Cell fusion between spleen cells and sp2/0 myeloma cells developed 1C3 hybridoma clone producing 1C3 monoclonal antibody (mAb). Hybridoma was injected into the mice to produce ascites, and further purified by caprylic acid/ammonium sulfate method. The mAb was of IgG3 subclass, and was specific to DA with high affinity (2.5 × 10^8^ L/mol). Moreover, western blot exhibited significant specificity to the DA antigens. Indirect competitive enzyme-linked immunosorbent assay (ic-ELISA) showed DA working range of 0.006–0.2 ng/mL. The IC_50_ was 0.03 ng/mL with low limit of detection (LOD) of 0.006 ng/mL. Average DA recovery from spiked shellfish extract was 100.56 ± 2.8% with the coefficient variation of 0.01–0.1%. Hence, mAb producing 1C3 hybridoma was successfully developed and could be used to detect DA in contaminated samples.

## 1. Introduction

Marine toxins are noxious, poisonous and hazardous substances, which can be found as short peptides, proteins or secondary metabolites. The toxins are produced by a range of phytoplankton taxa and other related microorganisms causing deadly diseases and lethal impacts on the health of marine mammals, birds and humans [1]. DA is a potent toxin, marine biotoxin, and hazardous to the human health. It is produced by the diatomic algal genus of *Pseudo-nitzschia* and other related species. DA is a characteristic hapten having molecular formula C_15_H_21_NO_6_, molecular weight of 311.3303 g/mol, contains three carboxyl groups for carbodiimide coupling, and one amino group [2,3]. Similarly, Ultraviolet Visible spectroscopy (UV-vis) analysis showed that DA exhibits visible absorbance at wavelength ranging 240–242 nm [2,3,4]. Furthermore, it is transmitted to the marine mammals and then to the humans via food chain by the consumption of contaminated seafood products primarily including species related to the shellfish [3,5].

DA was responsible for the poisoning incident in Canada in late 1987, and caused high brain toxicity in the effected individuals [3]. It is a glutamate analog, which acts as a potent excitatory neurotransmitter, and results in hippocampal excitotoxicity by increasing the neuronal calcium influx through kainate receptor activation. Severe neuronal damage in hippocampal CA3 and dentate gyrus regions have been observed in developing postnatal mice following intrauterine exposure to DA [3,6,7]. Moreover, the toxicity results in severe neural tissue loss compromising central nervous system leading to physiological disorders and memory impairment. Hence, the intoxication symptoms were named amnesic shellfish poisoning (ASP) because the victims suffered from memory loss [6]. DA toxin upon high intoxication causes severe tissue necrosis specifically destroying hippocampus [3,8]. Furthermore, it is also responsible for the disruption of multiple organ systems such as heart, brain, liver and kidney. The main seafood source identified for DA in the late 1987 incident was blue mussels (*Mytilus edulis*) [3]. Additionally, several other intoxication incidents have been documented in many coastal areas across the globe. These incidents reveal that DA is a highly prevalent marine toxin that is a source of persistent hazard to the human health via consumption of contaminated seafood [3,9]. The levels of DA determined to be unsafe for the human consumption is 20 µg/g detected in shellfish meat tissue and is an accepted standard internationally. Therefore, it signifies that detection and management of this potent life threatening toxin contamination in seafood samples is utmost vital for the safety of the human health [3,8].

The most frequently used instrumental detection methods for DA are liquid chromatography-tandem mass spectrometry (LC-MS/MS), liquid chromatography ultraviolet detection (LC-UV), biosensors, and immunoassays [3,10,11]. Instrument-based methods are sensitive and they can be used simultaneously for the analysis of multiples toxins, however, the drawbacks include equipment complexity, exhibit incompatibility, costly and prolonged when use for the real samples [12]. ELISA is an exceedingly sensitive immunoassay using enzyme-conjugated antibodies, with antigen or antibodies captured to a solid binding support such as ELISA plate [6,13,14,15]. The assay determines variations in enzymatic events relative to the analyte concentrations involved in the immune assay specifically designed for the detection of antigen or any substance [14,15]. The monoclonal antibody (mAb) based detection of toxins is rapid, specific, sensitive, low cost, and compatible with real samples. A limited number of immunoassays have been established for DA detection [6,16,17,18,19], but there are still some disadvantages such as higher LODs, higher working range and higher IC_50_, respectively. Ic-ELISA is a widely applicable antibody-based assay for the detection of toxins [20,21]. Therefore, a mAb against DA toxin has been produced in this study to detect the presence of DA in the real samples by establishing an improved ic-ELISA immunoassay.

## 2. Results

### 2.1. Analysis of DA-Protein Conjugates

**Figure 1:**
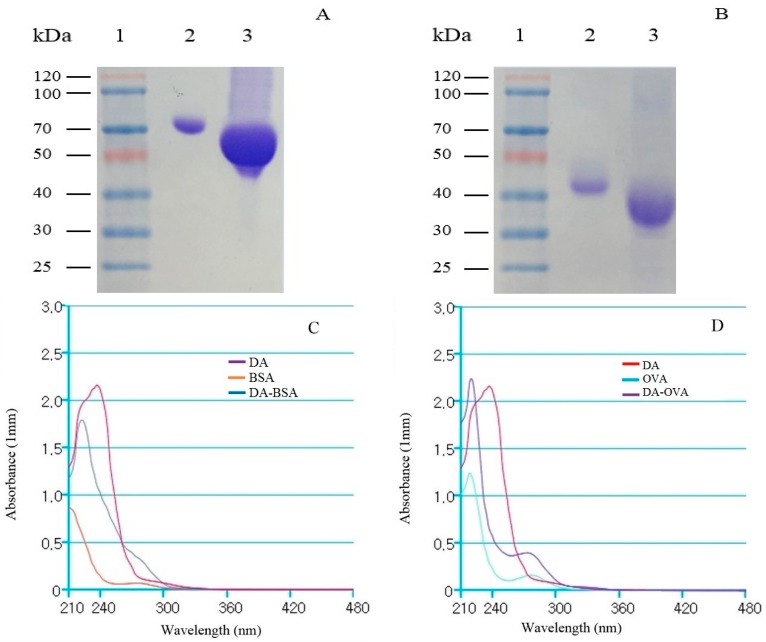
SDS-gel electrophoresis and UV-vis analysis of DA-protein conjugates. (**A**) Lane 1: Protein marker, Lane 2: BSA, Lane 3: DA-BSA conjugate; (**B**) Lane 1: Protein marker, Lane 2: OVA, Lane 3: DA-OVA conjugate; (**C**) UV-vis analysis of DA, BSA and DA-BSA conjugate; (**D**) UV-vis analysis of DA, OVA and DA-OVA conjugate. (BSA: bovine serum albumin, OVA: ovalbumin).

DA toxin is a non-immunogenic hapten, and therefore conjugated with carrier protein i.e., BSA to elicit immunogenic response in the mice and OVA as coating-antigen. Sodium dodecyl sulfate-polyacrylamide gel electrophoresis (SDS-PAGE) was used to assay successful DA-protein conjugation, and the result showed that DA-BSA and DA-OVA conjugates moved faster than carrier proteins alone, demonstrating that DA was successfully conjugated (Figure 1A,B). Ultraviolet spectral scanning curves were further obtained, and the shifts in wavelength were showed by DA-BSA (Figure 1C) and DA-OVA (Figure 1D) conjugates from DA, demonstrating successful conjugation.

### 2.2. Anti-Serum Analysis by Indirect Non-Competitive ELISA from Administered Mice

**Figure 2:**
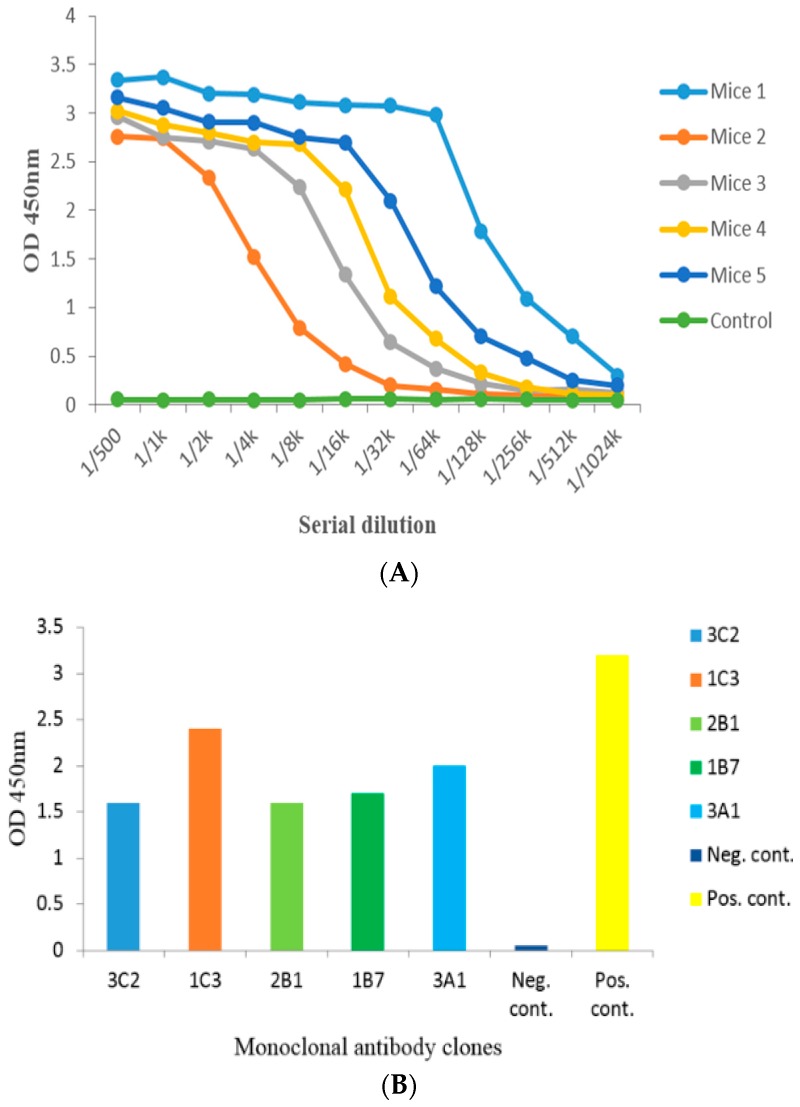
Serum titer assay and screening of positive hybridoma cells against DA toxin. (**A**) anti-DA serum titer of five mice by iELISA. Mice 1 showed highest anti-serum titer and selected for cell fusion; (**B**) Titer of supernatants of different hybridoma clones determined by iELISA.

Bicinchoninic acid assay (BCA) kit was used to evaluate the quantification of DA-protein coupling, and the concentrations of DA-BSA and DA-OVA were 6.405 and 5.55 mg/mL, respectively. The anti-serum titer of DA-BSA administered mice was tested by indirect non-competitive ELISA (iELISA), and mouse 1 demonstrated the highest anti-serum titer (1:1,024,000 *v*/*v*). These results showed successful immunogenic response induced by DA-BSA conjugates (Figure 2A). Hence, DA-BSA was used as immune antigen, and DA-OVA was used as coating-antigen in this study.

### 2.3. Cell Fusion and Screening of the Positive Clone

Spleen cells (splenocytes) were harvested from mouse 1 and fused with Sp2/0 myeloma cells. The supernatant of growing hybridoma cells was tested by iELISA, and positive hybridoma clones were screened. Five positive hybridoma clones were successfully obtained producing anti-DA mAb i.e., 3C2, 1C3, 2B1, 1B7, and 3A1, respectively (Figure 2B). In this study, the average fusion rate and the positive rate of the successful cell fusions was 87.50% and 14.93%, respectively (Table 1). Finally, a stable 1C3 hybridoma cell line producing 1C3 mAb (anti-DA mAb) was selected, and used for further studies.

### 2.4. Subclass and Chromosome Count of 1C3 Hybridoma Cell Line

**Figure 3:**
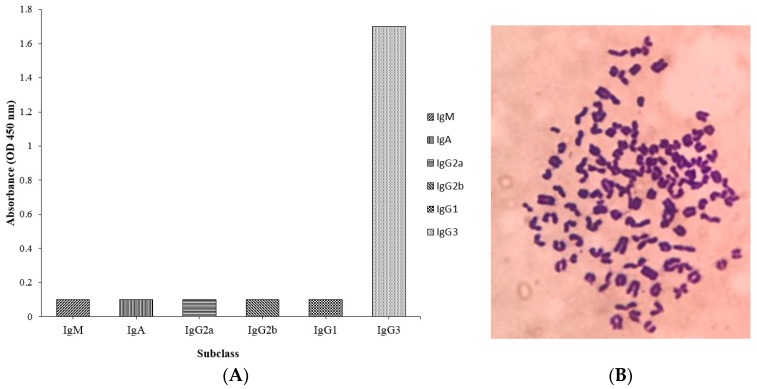
Subclass and chromosome count analysis of anti-DA hybridoma cell line. (**A**) Isotyping of 1C3 cell stably secreting 1C3 mAb by using the commercial isotyping kit for subclass analysis; (**B**) Chromosomes of 1C3 hybridoma cell analyzed by Geimsa staining and observed under inverted microscope.

The hybridoma cell line 1C3 against DA was analyzed for subclass using a commercial isotyping kit (IgM, IgG1, IgG2a, IgG2b, IgA, and IgG3). The results of the subclass analysis showed that the mAb producing 1C3 hybridoma cell had the IgG3 subclass (Figure 3A). The chromosome count analysis by Geimsa staining exhibited that the chromosome number of 1C3 hybridoma cell line was 102 ± 4 (Figure 3B) among the experiment replicates, and was produced by cell fusion between splenocytes with chromosome number 66 ± 4 and Sp2/0 myeloma cells with chromosome number 39 ± 1, respectively [22]. Therefore, the results indicated that the 1C3 hybridoma clone was produced by successfully fusing splenocyte and the Sp2/0 cell.

### 2.5. Production and Purification Analysis of the 1C3 mAb

**Figure 4:**
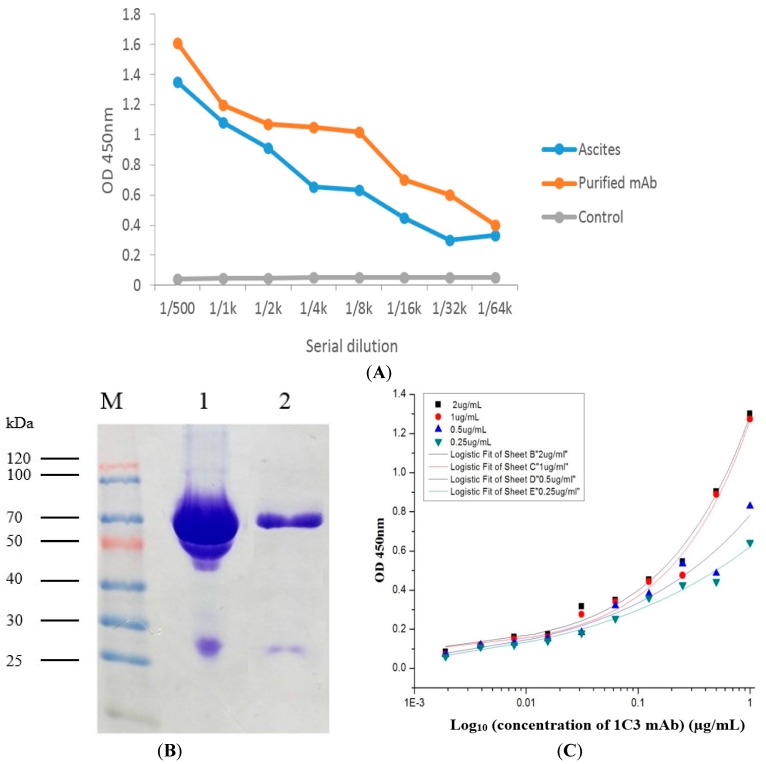
Purification, iELISA and affinity assay of the 1C3 mAb. (**A**) iELISA was used to determine the anti-DA activity of ascites and purified 1C3 mAb. The results showed that purified 1C3 mAb successfully captured DA antigen coated in iELISA; (**B**) Analysis of 1C3 mAb purification by SDS-gel electrophoresis. Lane M: protein marker, Lane 1: unpurified ascites fluid, Lane 2: purified 1C3 mAb; (**C**) The affinity result of the 1C3 mAb was based on different concentrations of the DA-OVA as coating antigen. The affinity result of mAb to the different concentrations of DA showed that the 1C3 mAb was a high affinity antibody.

Hybridoma cell clone 1C3 was injected inraperitoneally (i.p.) into the primed (pristane) mice. Ascites fluid was harvested and the 1C3 mAb was significantly purified by caprylic acid/ammonium sulfate method and subsequently confirmed by iELISA for anti-DA activity of the ascites and purified mAb compared with the control (Figure 4A). The results demonstrated that the 1C3 mAb had high anti-DA activity and successfully captured DA antigen coated in iELISA. Moreover, mAb showed high antibody titer (1:64,000 *v*/*v*), and hence successfully purified. The concentration of the 1C3 mAb was determined by BCA kit after purification and was 1.63 mg/mL. Moreover, the mAb was assayed by SDS-gel electrophoresis. The results from the SDS-gel analysis revealed that the heavy chain of the mAb was at 70 kDa and the light chain was at 27 kDa, respectively (Figure 4B). Therefore, indicating that the 1C3 mAb was successfully purified from the ascites and could be used for further experiments.

### 2.6. Affinity Analysis of 1C3 mAb

The iELISA was used for the affinity analysis of the 1C3 mAb was based on the different concentrations of the DA antigen (2, 1, 0.5, and 0.25 µg/mL) coated as DA-OVA. The data obtained from the affinity analysis and the affinity constant was calculated by data analysis software Microcal Originpro 9.1. The affinity results demonstrated that the mAb secreted by the 1C3 hybridoma clone was sensitive to the DA antigen, and the average affinity constant calculated for 1C3 mAb was 2.5 × 10^8^ L/mol (Figure 4C). Hence, indicating that the mAb obtained from the 1C3 hybridoma clone was a high affinity antibody produced against the DA toxin and could be used for further studies.

### 2.7. Specificity of 1C3 mAb

**Figure 5:**
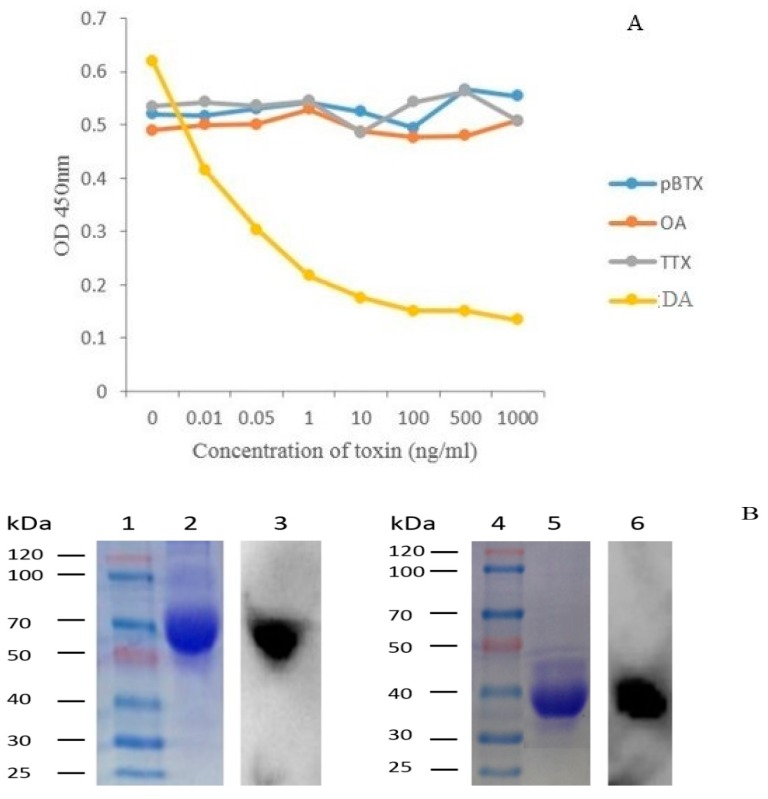
Specificity results of 1C3 mAb. (**A**) The specificity was determined by competent ELISA, showing no cross-reactivity with other related marine toxins; (**B**) Western blot analysis was carried out to check specificity of 1C3 mAb against antigens DA-BSA and DA-OVA respectively. Lane 1 and 4: Protein Marker. Lane 2 and 5: SDS-PAGE of DA-BSA and DA-OVA respectively. Lane 3 and 6: Western blot results of anti-DA mAb against DA-BSA and DA-OVA antigens.

The antigenic specificity analysis of the 1C3 mAb was evaluated by competitive inhibition ELISA and western blot respectively. The results in Figure 5A demonstrated that the 1C3 mAb was significantly specific to DA, and no cross-reactivity to other marine toxins including pBTX, OA, and TTX was observed. Western blot results exhibited distinct bands against target antigens (Figure 5B), indicating that the 1C3 mAb specifically recognized DA-BSA and DA-OVA antigens, respectively. All these results demonstrated that the 1C3 mAb was highly specific to DA.

### 2.8. Preparation of the Standard Curve

**Figure 6:**
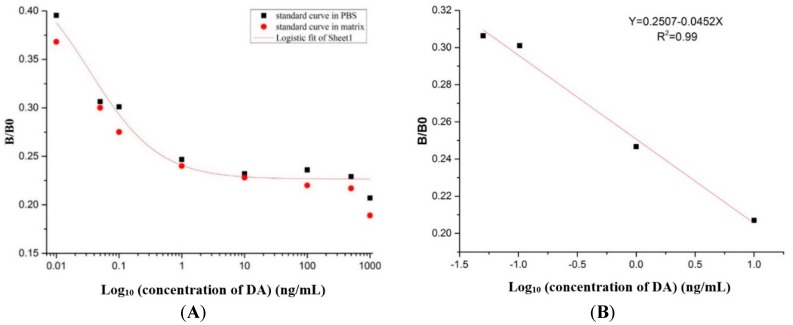
Standard curves for DA detection. (**A**) Standard curve was obtained by plotting (B/B0) against DA concentration. The equation was *y* = 0.447/(1 + (*x*/0.0349)^0.795^), and the correlation coefficient (R^2^) was about 0.98; (**B**) The linear portion of standard curve. *y* = 0.2507 − 0.0452*x* with the correlation coefficient (R^2^) 0.99.

The standard curve and recovery test of the 1C3 mAb was analyzed by competitive inhibition ELISA. The standard curve was prepared and the data was analyzed by Microcal OriginPro 9.1 to test the relationship of the concentration of DA with its inhibition. The standard curve was analyzed by the logistic curve and the equation was *y* = 0.447/(1 + (*x*/0.0349)^0.795^), and the correlation coefficient (R^2^) was about 0.98 (Figure 6A). The linear equation was *y* = 0.2507 − 0.0452*x* with the correlation coefficient (R^2^) 0.99 (Figure 6B). In the present study, the half inhibitory concentration (IC_50_) was 0.03 ng/mL, and the linear range of detection was 0.006–0.2 ng/mL with a lower LOD of 0.006 ng/mL.

Moreover, the matrix interference was analyzed by ic-ELISA based on the different concentration of DA toxin that is usually found in shellfish seafood products. To test the matrix affect, the PBS and sample matrix was artificially contaminated with DA and used for the determination of the matrix effect. The standard curves were prepared in the PBS and the matrix based on the results of ic-ELISA (Figure 6A). Therefore, the matrix interference was reduced by 100-fold dilution, that means it was suitable for the establishment of the ic-ELISA assay, and therefore was appropriate for the determination of DA in real samples.

### 2.9. Analysis of Recovery Test and Sample Detection by ic-ELISA Using 1C3 mAb

Recovery test was carried out to assess the quality of the established ic-ELISA, Non-contaminated shellfish extract was diluted properly to minimize the matrix effect and was spiked with DA at various concentrations (0.5, 5, 100, 1000 ng/mL) (Table 2). The results demonstrated that the recovery range was from (95.03 ± 5.4)% to (106.18 ± 1.7)% with an average recovery of (100.56 ± 2.8)%, and its coefficient of variation (CV) was ranged from 0.01 to 0.1% with an average CV of 0.03% (Table 2). Real shellfish samples (abalone, sea mussel, fresh oyster, giant clam, king scallop, razor clam, fresh water mussel and triangular clam) were randomly purchased from the seafood market for sample analysis. After extraction, the samples were assayed independently in duplicates to check the working of the established ic-ELISA for DA detection in complex matrix. The results showed that four samples from all shellfish samples were DA positive with levels under 30 ng/mL (120 ng/g), and the extracts of razor clam and giant clam showed the highest levels of DA at 6.70 ng/mL (26.80 ng/g) and 2.89 ng/mL (11.56 ng/g), respectively. Moreover, extracts of abalone (0.76 ng/mL) and fresh oyster (0.02 ng/mL) exhibited least detection, demonstrated DA levels lower than 1.0 ng/mL (4 ng/g). Similarly, all other shellfish samples were DA free with no contamination compared with the control in ic-ELISA at OD 450 nm using 1C3 mAb in the study (Table 3).

## 3. Discussion

DA is a potent toxin, severe health hazard to the humans, causes neurotoxicity in CNS and results in several disorders in multiple organ systems such as heart, brain, liver and kidney [3]. DA is a characteristic hapten with low molecular weight. Therefore, it is unable to elicit an immunogenic response in the mice to develop a mAb. Thus, it was essential to conjugate the DA antigen with larger carrier proteins to produce immune response and production of the mAb [15]. Structurally, there are several functional groups present in the DA that are available for the conjugation with carrier protein. Bovine serum albumin (BSA, MW 67,000) has 59 lysine (NH2) functional groups available for the conjugation [23], and OVA (MW 45,000) has 20 lysine (NH2) functional groups, respectively. In the present study, we applied the modified conjugation method from the previous study of DA with BSA and OVA [16].

The conjugation was carried out through the formation of amide bonds using EDC and its coupling dehydrating agent NHS comparing to the complexed conjugation method [24]. The conjugation of DA with carrier proteins was carried out in the borate buffer, and the results indicated that functional groups of the carrier proteins and DA have been successfully coupled for the formation of DA-BSA and DA-OVA. In SDS-gel electrophoresis, the migration speed of conjugates was faster than that of the carrier proteins alone, because the conjugated products carried more negative charges from DA, therefore, the net charge of the products became more negative compared to the carrier proteins [25], Moreover, UV-vis analysis showed that superposition properties of the conjugated products shifted in wavelengths from 242 nm of standard DA to 225 nm, indicating successful conjugation of DA with the carrier proteins, respectively [26]. Therefore, these results exhibited successful development of both conjugates. Moreover, DA-BSA conjugate was used for administering the mice and DA-OVA was used for coating antigen. After administering the mice, DA-BSA produced high anti-serum titer. The results indicated that DA was successfully conjugated with the carrier proteins and it further helped in signifying the potential use of the conjugates for animal administration and antibody screening [27].

Similarly, high anti-serum titers were induced by DA-BSA antigen in the Balb/c mice, and then cell fusion in the presence of PEG-1450 was carried out between the splenocytes harvested from the administered mice and the Sp2/0 cells. The cell fusions were successful demonstrated by the average fusion rate (87.50)% and the average positive rate (14.93)%, respectively. Therefore, based on the previous studies, the average cell fusion rates were appropriate for the production and effective screening of the hybridoma cell clones stably producing anti-DA mAb [28,29]. The successful screening resulted in the production of 1C3 positive hybridoma clone, which was injected i.p. into the primed Balb/c mice. The ascitic fluid was harvested containing 1C3 mAb, and caprylic acid/ammonium sulfate method was used to purify the mAb. After purification of the mAb, iELISA was used for the positive 1C3 antibody activity of the ascites and 1C3 mAb captured against DA antigen in the conjugates, and high antibody titers showed successful purification of the mAb. The SDS-gel electrophoresis showed significantly distinct bands of the target 1C3 mAb in relation to the ascites, and the results were in good agreement with our previous study of antibody purification [29]. Consequently, the purification results from SDS-gel showed that the 1C3 mAb was successfully purified and could be used for further studies.

Moreover, the specificity of the mAb secreted from 1C3 hybridoma clone was determined by the competitive inhibition ELISA and western blot. The ELISA results demonstrated that the mAb was highly specific to the DA antigen. Furthermore, western blot analysis was also used to analyze the antigenic specificity of 1C3 mAb, and the results confirmed the specificity of 1C3 mAb against the DA antigens [30]. Moreover, the affinity results of the mAb to DA demonstrated in high affinity and the affinity constant was 2.5 × 10^8^ L/mol. The results further showed that the affinity range was good. A previous study about antibody affinity described that a mAb within 10^7^ to 10^12^ L/mol affinity could be used and had better potential for the development of immunoassay and other applications [31]. Hence, the mAb produced in this study was highly specific and had good affinity against DA antigen. Likewise, these results indicated that the antibody secreted by 1C3 was a good affinity mAb, had high specificity to DA antigens and could be used for detection of DA in shellfish samples [16,17,19].

Furthermore, from the results of the established ic-ELISA standard curve, the calculated IC_50_ was 0.03 ng/mL and was considerably lower than IC_50s_ obtained in the previous ELISA studies i.e., 0.58 ng/mL [19], 156 ng/mL [16], and 10 ng/mL [17], respectively. Similarly, the working range of antigen detection was 0.006–0.2 ng/mL, which was described as the concentration of DA from 20% inhibition to 80%, and was significantly lower than the working ranges reported in the previous studies such as 10–260 ng/mL [32], 0.1–15 ng/mL [33], 0.15–10 ng/mL [34], and 4–60 ng/mL [35], respectively. Likewise, the lower LOD obtained in the present study was 0.006 ng/mL, which was considerably lower than the LODs reported in the previous studies i.e., 4 ng/mL [17], 3 ng/mL [35], and 15 ng/L [36], respectively. The extracted shellfish samples were tested for the working of ic-ELISA established in this study in complex matrix, and four samples were DA positive with lower levels of DA present under 120 ng/g. The standard levels of DA determined to be unsafe for human consumption is 20 µg/g in shellfish meat tissue. Therefore, the results validated the working of ic-ELISA in complex matrix, showed insignificant levels of DA toxin found in the shellfish samples, and hence these samples were not contaminated and safe for the human consumption [8]. The ic-ELISA established in this study showed higher sensitivity demonstrating lower DA level in real shellfish samples (4 ng/g) compared with lower sensitivity exhibited previously by instrumental detection methods i.e., LC-MS/MS (9 ng/g) [37], LC-UV (6 ng/g) [38], respectively. Therefore, the results showed that the 1C3 mAb secreted by 1C3 hybridoma could be used to develop an ic-ELISA kit for the detection of DA. The recovery test on spiked shellfish extract showed a (100.56 ± 2.8)% mean recovery rate with a 0.03% average coefficient of variation, signifying that the method was appropriate for DA detection in the real shellfish samples [34]. Hence, the ic-ELISA established in the present study together with high affinity, high specificity, high sensitivity, low LOD and low IC_50_ was considerably feasible to detect DA toxin in the seafood shellfish samples. Taken together, this 1C3 mAb from 1C3 hybridoma would offer a base information to develop more sophisticated immunoassay kits, to evaluate the risk of DA contamination in seafood shellfish products, and will assist to prevent the toxicity threats from similar hazardous substances to the fragile marine wildlife and human health.

## 4. Conclusions

DA is a biotoxin that causes ASP in humans. Therefore, 1C3 hybridoma was developed producing 1C3 mAb against DA. The mAb had high affinity and high specificity to DA antigens. Ic-ELISA sensitive immunoassay was established for the toxin recovery and sample analysis. Standard curve showed low IC_50_, low LOD and feasible working range for DA detection in the real shellfish samples. The immunoassay revealed very low levels or no toxin in the samples compared with standard DA levels unsafe for human consumption. Thus, the samples were non-contaminated and safe for human consumption. The ic-ELISA test kit established in this study using 1C3 mAb provides an accurate, flexible and cost effective method for measuring DA in real shellfish samples. Moreover, the test kit could be used for DA detection in shellfish seafood products for monitoring marine wildlife and the human health.

## 5. Materials and Methods

### 5.1. Materials, Animals and Cell Line

DA, 1-ethyl-3-(3-dimethylaminopropyl) carbodiimide hydrochloride (EDC), *N*-hydroxysuccinimide (NHS), dimethyl sulfoxide (DMSO), methanol, polyethylene glycol 1450 (PEG-1450), phosphate-buffered saline (PBS, pH 7.4), borate buffer (0.085 M), ovalbumin (OVA), bovine serum albumin (BSA), Freund’s complete and incomplete adjuvant, pristane (2,6,10,14-Tetramethylpentadecane), HAT medium, horseradish peroxidase (HRP)-conjugated goat anti-mouse secondary antibody, polyethylene glycol (PEG-1450), IgG1, IgG2a, IgG3, IgM, IgA mouse mAb isotyping kit, hypoxanthine-thymidine (HT) medium, and RPMI-1640 were acquired from Sigma-Aldrich Chemical (St. Louis, MO, USA). The murine myeloma cell line Sp2/0 was stocked in liquid nitrogen at our laboratory. All Balb/c mice (female, six to eight-weeks old) used in the present study were obtained from the Wushi animal laboratory (Shanghai, China). All other chemical reagents used in the current study were acquired commercially in China and were of chemical grade.

### 5.2. Ethical Statement and Animal Care

All the studies related to the animals were conferring to the Committee for animal Ethics of Fujian Agriculture and Forestry University (FAFU) in China (C1017/23.12.2014). The mice were housed in a properly designed and well planned animal room with each animal cage containing three mice. Furthermore, the room was provided with favorable animal housing conditions including optimal temperature at 24 ± 2 °C and humidity at optimal range of 50–60%. Moreover, the animals were provided clean drinking water and Forti-Diet commercial pellet food for feeding. Additionally, regular health inspection of the mice and hygiene of the animal room was assured. All the experiments were performed in replicates to show the biological and measurement variability, respectively.

### 5.3. Synthesis of DA-Protein Conjugates and Analysis

DA-protein conjugates were prepared by active aster method using EDC, NHS and borate buffer as previously described with some modifications [16]. DA-BSA and DA-OVA conjugates were evaluated by SDS-PAGE and UV-vis [29,39]. The SDS analysis was carried out by mixing 10 µL conjugates and 10 µL SDS loading buffer and loaded to SDS gel (for 60 min at 200 V). After running electrophoresis, the gel was subjected to staining for 3 h at 60 °C with Coomassie blue R-250 and was properly washed with de-stain solution until the distinct protein bands were observed. The UV-vis of DA, carrier proteins and conjugates were determined by UV spectrophotometer at the wavelength of 200–500 nm respectively [4]. The concentration of DA-protein conjugates was determined by the BCA assay [40]. DA-BSA was used for administering mice and DA-OVA was used as coating antigen in ELISA, respectively.

### 5.4. Mice Administration and iELISA Assay of Anti-Serum

Five Balb/c mice were administrated primarily with DA-BSA conjugates (200 µg/mL) added with equivalent volume of Freund’s complete adjuvant at several sites subcutaneously. Booster injections of DA-BSA (100 µg/mL) added with equivalent volume of Freund’s incomplete adjuvant were given at two-week intervals. After five times administration, blood from each mice was harvested sequentially from the tail vein and individual anti-sera were assayed by iELISA [15]. Briefly, 96-well ELISA plate was coated with DA-OVA (100 µL/well), and incubated overnight at 4 °C. Plate was washed three times with PBS, blocked with PBSM (5% skim milk powder in PBS, 200 µL/well), and incubated for 2 h at 37 °C. Later, plate was washed three times with PBS, PBST (0.5% Tween 20 in PBS). Mice serum was added to the plate, and incubated for 2 h at 37 °C. After sufficient washing, goat anti-mouse IgG-HRP (1:8000, 100 µL/well) was added, and incubated for 1 h at 37 °C. After washing, a chromogenic reporter i.e., TMB (100 µL/well) was added, incubated for 15 min at 37 °C, and 2 M H_2_SO_4_ (50 µL/well) was added to stop the reaction mixture, a yellow reaction product was formed upon acidification that was measured at 450 nm by microplate reader [29,41].

### 5.5. Cell Fusion and Screening of Anti-DA mAb

Hybridoma cell line was developed against DA based on a standard modified method [29,42]. The anti-serum of the five mice was tested by iELISA, the mouse with highest anti-DA antibody titer was chosen and administered i.p. with DA-BSA 3 d prior the cell fusion. The splenocytes from administered mice were harvested and fused with Sp2/0 myeloma cells (grown in three cell culture dishes) at relative ratio at 1:10 in presence of PEG-1450 (1 mL) added dropwise [15,43]. Hybridoma cells were thoroughly cultured, HAT selected, and subsequently screened for anti-DA mAb by iELISA. Sub-cloning was carried out three times by limiting dilution method for subsequent screening of positive hybridoma clone and expanded [29].

### 5.6. Subclass and Determination of Chromosome Count of 1C3 mAb Producing Hybridoma

The subclass analysis of the positive hybridoma cells was carried out after subsequent sub-cloning according to the previously described method [29] with some modifications. Giemsa staining analysis [44] was used for the determination of chromosome count for positive hybridoma. The chromosome number of the respective positive hybridoma was determined subsequently after slide preparation in replicates and observed under inverted microscope [29].

### 5.7. Ascitic Production, Purification and Analysis of Purified 1C3 mAb

The 1C3 mAb mass production was carried out by priming three well grown and healthy Balb/c mice by administering pristane (500 µL). After 7 d, the primed mice were administered i.p. by hybridoma cell line 1C3. The developed ascites were harvested and centrifuged at 10,000× *g* for 10 min, and caprylic/ammonium sulfate method [15,45,46] was used for mAb purification with minor modifications. After purification, SDS-PAGE was used for the analysis of purified mAb [39], and the experiment was repeated three times for obtaining distinct bands of light and heavy chain of purified mAb. BCA kit [29] was used to test the concentration of the purified mAb in triplicates.

### 5.8. Affinity Analysis and Specificity of 1C3 mAb

The 1C3 mAb affinity was assessed by the previously published method [29] with some changes. DA-OVA as the coating antigen was coated at serially diluted concentrations (0.25, 0.5, 1, 2 µg/mL) overnight at 4 °C, and experiment was performed in triplicates. The remaining steps were the same as iELISA. Data was evaluated using data analysis software Microcal OriginPro 9.1, and the affinity constant of anti-DA mAb was analyzed by method [47] described before. The antigenic specificity and cross-reactivity of 1C3 mAb were done by our previously described study [48]. Several marine toxins other than DA such as *Brevetoxin* (Pbtx), *Okadaic acid* (OA) and *Tetrodotoxin* (TTX) were used as competitor antigens for 1C3 mAb in ic-ELISA carried out in triplicates. Western blot specificity analysis was also used to identify the antigenic specificity of the 1C3 mAb [30], distinct blot was obtained after repeating the experiment three times.

### 5.9. Development of ic-ELISA

The ic-ELISA was developed for the establishment of standard curves, recovery test and detection of DA in the real samples. The ic-ELISA was established conferring to the previously described method [28] with minor modifications. The optimal concentrations of the DA-OVA coating antigen was developed, coated in the ELISA micro well plates in triplicates and incubated overnight at 4 °C. Washed three times by PBST and by PBS subsequently. After washing, blocked by 5% PBSM (200 µL/well), and kept at 37 °C for 2 h. Later, washed with PBST and PBS respectively, equivalent volume of the 1C3 mAb and the free DA toxin was reacted at various concentrations (0, 0.01, 0.05, 0.1, 1, 5, 10, 100, 1000 ng/mL) in triplicates. After proper mixing, incubated at 37 °C for 30 min. Later, the reaction mix were transferred to the ELISA plates (100 µL/well) with each standard concentration, and incubated at 37 °C for 1 h. The next phases were the same as iELISA.

### 5.10. Preparation of the Standard Curve and the Recovery Test

The shellfish extract was contaminated artificially with DA toxin and was analyzed using ic-ELISA described above. The working range to detect DA was described as the concentration of DA toward from 20 to 80% inhibition of the maximum absorbance [3]. Inhibition concentration of DA toxin with respect to 1C3 mAb was assessed by Microcal OriginPro 9.1 [29]. After extraction with previously described method [49], the shellfish extract was spiked with various concentrations of the DA toxin (0.5, 5, 100 and 1000 ng/mL) in triplicates. The matrix effect was determined after developing and comparing the standard curve in the matrix and the PBS, respectively. Later, the matrix interference was reduced by diluting the sample extract based on the previous study, in triplicates with minor modifications. The standard curve was used for analyzing and determining the recovery tests. Data in replicates was used for the determination of the coefficient variation (CV) of the recovery tests [29]. Real shellfish samples were independently assayed for the working of ic-ELISA established described above in this study for DA detection in complex matrix.

## Figures and Tables

**Table 1:** Cell fusion rates and screening of positive hybridoma clones.

Plates No.	Fusion Rate (%)	Positive Rate (%)	Positive Hybridoma Clones *
1	90.63% (87/96)	14.58% (14/96)	1B7, 1C3
2	88.54% (85/96	17.71% (17/96)	2B1
3	83.33% (80/96)	12.50% (12/96)	3A1, 3C2
Average	87.50%	14.93%	

* Five anti-DA positive hybridoma clones with higher fusion and positive rates were screened after successive cell fusions from five 96-well plates.

**Table 2:** Accuracy of ic-ELISA evaluated by DA recovery from spiked shellfish extract.

Spiked Level (ng/mL)	Measured Concentration (ng/mL)	Recovery (%)	CV (%)
0.5	0.53 ± 0.01	106.18 ± 1.7	0.02
5	5.25 ± 0.2	104.99 ± 3.4	0.03
100	95.03 ± 5.4	95.03 ± 5.4	0.1
1000	960.52 ± 7.8	96.05 ± 0.8	0.01
Average		100.56 ± 2.8	0.03

± Represents the average deviation from the mean that was analyzed in duplicates. Data were given as the average value. The coefficient of variation (CV) was described as the ratio of the standard deviation to the average in the spiked recovery test.

**Table 3:** The detection of DA toxin in the real samples by ic-ELISA.

Shellfish Samples	OD 450 nm	Detection Results (ng/mL) ^a^
Control PBS (B0)	0.44 ± 0.03	ND ^b^
Abalone	0.28 ± 0.003	0.76 ± 0.5
Sea mussel	0.44 ± 0.01	ND
Fresh oyster	0.32 ± 0.01	0.02 ± 0.01
Giant clam	0.26 ± 0.01	2.89 ± 2.5
King scallop	0.41 ± 0.1	ND
Razor clam	0.25 ± 0.01	6.7 ± 6.4
Fresh water mussel	0.44 ± 0.04	ND
Triangular clam	0.41 ± 0.01	ND

^a^ One milliliter extract solution contains 0.25 g of shellfish tissue; ^b^ ND means not detected. ± represents the value of the average deviation from the mean that was analyzed in duplicate. Data were given as the mean value.
